# Nanomaterials and hepatic disease: toxicokinetics, disease types, intrinsic mechanisms, liver susceptibility, and influencing factors

**DOI:** 10.1186/s12951-021-00843-2

**Published:** 2021-04-16

**Authors:** Ting Sun, Yiyuan Kang, Jia Liu, Yanli Zhang, Lingling Ou, Xiangning Liu, Renfa Lai, Longquan Shao

**Affiliations:** 1 Foshan Stomatological Hospital, Foshan University, Foshan, 528000 China; 2 Stomatological Hospital, Southern Medical University, Guangzhou, 510280 China; 3 Medical Center of Stomatology, The First Affiliated Hospital, Guangzhou, 510630 China

**Keywords:** Nanomaterials, Hepatic disease, Mechanisms, Susceptible individuals, Toxicokinetics

## Abstract

The widespread use of nanomaterials (NMs) has raised concerns that exposure to them may introduce potential risks to the human body and environment. The liver is the main target organ for NMs. Hepatotoxic effects caused by NMs have been observed in recent studies but have not been linked to liver disease, and the intrinsic mechanisms are poorly elucidated. Additionally, NMs exhibit varied toxicokinetics and induce enhanced toxic effects in susceptible livers; however, thus far, this issue has not been thoroughly reviewed. This review provides an overview of the toxicokinetics of NMs. We highlight the possibility that NMs induce hepatic diseases, including nonalcoholic steatohepatitis (NASH), fibrosis, liver cancer, and metabolic disorders, and explore the underlying intrinsic mechanisms. Additionally, NM toxicokinetics and the potential induced risks in the livers of susceptible individuals, including subjects with liver disease, obese individuals, aging individuals and individuals of both sexes, are summarized. To understand how NM type affect their toxicity, the influences of the physicochemical and morphological (PCM) properties of NMs on their toxicokinetics and toxicity are also explored. This review provides guidance for further toxicological studies on NMs and will be important for the further development of NMs for applications in various fields.
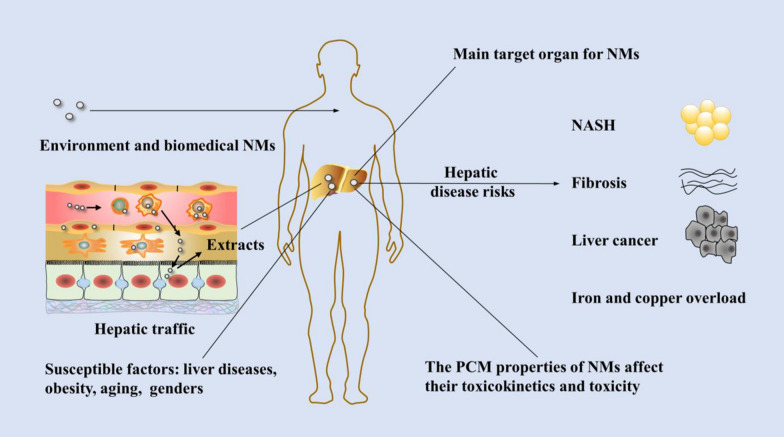

## Background

With the development of nanotechnology, nanomaterials (NMs) are increasingly being used in various industrial, agricultural and commercial applications—e.g., as additives in food, sunscreens, cosmetics, pharmaceutical products, agricultural products and diesel fuel [1–3]. In healthcare and the life sciences, NMs also have different medical uses (e.g., as drug delivery systems, antibacterial agents, magnetic resonance imaging (MRI) agents, and hyperthermia treatment media) and biotechnological applications (e.g., as biosensors) [4]. The widespread use of NMs has raised concerns that human exposure to NMs may introduce potential risks to the human body and environment. The NMs currently used in various fields cover a broad spectrum, ranging from liposomes, polymers, micelles, nanocrystals, and protein-based NMs to inorganic NMs. Generally, organic NMs are considered less toxic than inorganic NMs because they are not biopersistent and are more likely to be metabolized and cleared from the body [4]. Thus, the literature on nanotoxicity has focused mainly on the toxicity of inorganic NMs.

The liver is the primary organ involved in the metabolism and detoxification of toxins and xenobiotics and is a major target organ for NMs. Consequently, liver impairments—e.g., oxidative stress, inflammatory stress, liver dysfunction, DNA damage, steatosis, fibrosis, and cell death—caused by NMs have been observed in numerous studies [5–8]. However, the hepatotoxic effects of NMs have not been linked to liver disease, and the intrinsic mechanisms have not been well demonstrated. Additionally, NMs exhibit varied toxicokinetics and induce enhanced toxic effects in susceptible livers; however, this issue has been ignored in recent toxicity studies. Therefore, this review discusses possible NM-induced liver diseases and the intrinsic mechanisms based on the current understanding of NM toxicokinetics and considers the contribution of potential susceptibility factors to provide guidelines for the biosafety of NMs. To understand how NM type affect their toxicity, the influences of the PCM properties of NMs on their toxicokinetics and toxicity are also explored.

## Characteristics of the liver

The liver is a solid organ comprising mainly hepatocytes and less abundant cells. The less abundant cells include monocytes, Kupffer cells (KCs), liver sinusoidal endothelial cells (LSECs), hepatic stellate cells (HSCs), biliary epithelial cells (e.g., BECs and cholangiocytes), resident immune cells (i.e., natural killer cells, dendritic cells and lymphocytes), hepatic progenitor cells (HPCs), and circulating blood cells and immune cells in transit through the liver. KCs, the major components of the mononuclear phagocyte system (MPS), the largest population of resident macrophages in the body, account for 15% of the total liver cells and perform a janitorial function, protecting hepatocytes through the removal of xenobiotics and cellular debris via cellular phagocytosis and phagolysosomal digestion [9]. The hepatic barrier comprises LSECs and the extracellular matrix (ECM). The ECM is a network of collagen, proteins, and elastic fibers that provide the structural integrity to the tissue. LSECs are highly specialized endothelial cells, characteristic of a discontinuous architecture, meaning that fusion of the luminal and abluminal plasma membrane occurs at sites other than cell junctions, in areas called ‘fenestrae’. HSCs are involved in forming basement membrane-like structures via the secretion of laminin, proteoglycans, and type IV collagens. Hepatocytes, which exist under the LSEC layer, constitute 70%–80% of all liver cells and are responsible for maintaining liver functions.

**Fig. 1:**
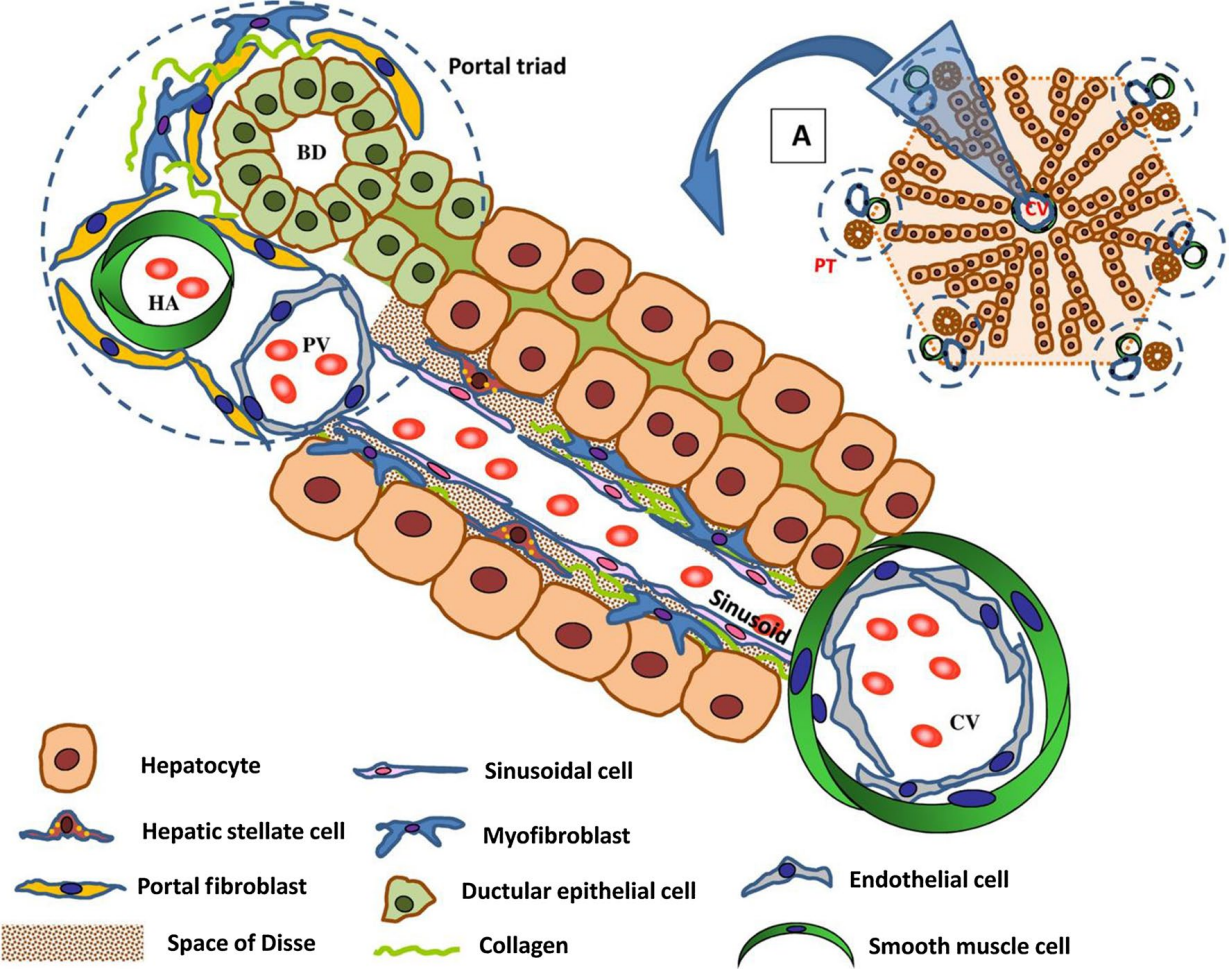
Schematic of the liver structure and hepatic cells. The liver comprises lobules, representing hepatic functional units (panel A). Each lobule is intersected by a central vein (CV), from which hepatocyte cords radiate towards portal triads (PTs) containing three different structures: bile ducts (BD), a hepatic artery (HA) and a portal vein (PV). Hepatocyte cords are separated by sinusoids, which are blood vessels lined by specialized fenestrated endothelial cells (liver sinusoidal cells). Hepatic stellate cells (HSCs) are located in the space of Disse, and portal fibroblasts (PFs) are located in the portal triad areas. Ductal epithelial cells line the bile ducts. Reprinted with permission from [202]. Copyright 2017 Wiley

A schematic diagram of the liver structure and hepatic cells is shown in Fig. 1.

## Characteristics of NMs

Normally used inorganic NMs can generally be classified as metal or metal oxide NMs (e.g., Au-, Ag-, ZnO-, TiO_2_-, and CuO-NPs) or non-metal NMs (e.g., carbon nanotubes (CNTs), black phosphorus (BP) NMs, and Si-NPs). NMs can also be classified by their structure and shape into quantum dots (QDs), nanotubes (NTs), nanowires, nanorods (NRs) and nanobelts.

### Metal NMs

ZnO-NPs are among the most commonly used NMs in industrial, biomedical and dental applications. For example, ZnO-NPs are commonly used as additives in electronic components because of their semiconductor properties and are considered important physical UV filters in sunscreens due to their very high UV-protection capacity. ZnO-NPs also exhibit fair antimicrobial, antifungal and anti-tumor cell properties because of their high surface-to-volume ratio and ability to destroy the cell integrity, release Zn^2+^ ions and stimulate reactive oxygen species (ROS) formation [10]. However, studies have suggested the toxic effects of both solid ZnO-NPs and dissolved Zn^2+^ on normal cells [11].

Recently, TiO_2_-NPs have been widely applied in industrial and consumer products—e.g., cosmetics, paints, glass, electronic devices, and food additives—because of their high catalytic activity, high stability and resistance to corrosion. However, TiO_2_ is considered a human carcinogen by inhalation (Category 1B) by the Risk Assessment Committee of the European Chemicals Agency (ECHA), and TiO_2_-NP toxicity is closely related to the release of Ti^2+^ [12]. Furthermore, the French Agency for Food, Environmental and Occupational Health and Safety (ANSES) banned the application of TiO_2_ as food additives (E171) because of its genotoxic potential [13].

Because of their unique physiochemical properties, copper (Cu)-based NPs—e.g., Cu-NPs, CuO-NPs and Cu_2_CO_3_(OH)_2_-NPs—are widely applied in technical fields, such as in wood protection, electronics, catalysts, antimicrobial supplements in poultry feed and intrauterine contraceptive devices, and are taken up by humans via inhalational and oral routes (hand-to-mouth transfer during installation) [14]. Under overload conditions, copper is potentially toxic because of its redox activity and high affinity towards thiols and proteins or enzymes.

Ag-NPs are widely used in food additives, food packaging, textiles, electronics, cosmetics, household appliances, medical devices, room sprays and water disinfectants because of their attractive antibacterial and plasmonic properties. However, no silver-based nanocarriers have been approved by the FDA for the systemic delivery of other bioactive agents [15]. The surface of Ag-NPs can be oxidized by O_2_ and other molecules in the environment and biological systems, resulting in the release of Ag^+^, a known toxic ion. Ag-NPs can lead to physical damage to the cell membrane and ROS generation, resulting in dysfunction of key cellular components following the release of Ag ions [16].

### Non-metal NMs

Carbon NMs include 0D fullerene, carbon dots, nanodiamonds, graphene QDs, 1D CNTs, 2D graphene and its derivatives, and graphitic carbon nitride. Carbon NMs are widely used in biomedicine, energy and sensing because of their excellent electronic and optical properties, good biocompatibility, fair surface function and high reactivity. However, because of their light weight and small size, they are prone to aerosolization, leading to inhalation and potential toxic effects [17].

Si-NPs are widely applied in industry as food additives, beverage ingredients, drug and vitamin excipients, and cosmetic product additives. Mesoporous Si-NPs have striking characteristics for application as drug carriers because they can adsorb and release drug molecules. However, Si-NPs can absorb toxic agents on the cell surface, increasing their toxicity potential [18].

## Toxicokinetics of NMs

Recently, the toxicokinetics of NMs has become an important part of nanotoxicological studies. In this section, the toxicokinetics of NMs is discussed.

### Administration routes and distribution of NMs

NMs enter the liver through the blood circulation. NMs enter the blood circulation directly or indirectly by passing through normal physiological barriers, such as the air-blood barrier (ABB) and gastrointestinal tract (GIT) barrier. The liver uptake ratio of NPs (from high to low) administered via different routes is as follows: intravascular, inhalation, and oral administration.

After entering the blood circulation, intravenously injected NMs are primarily captured by the MPS. As a filtration organ containing cells in the MPS with a fenestrated vasculature and high populations of committed phagocytes, the liver rapidly captures a large amount of intravascularly administered NMs after administration. Approximately, 30%–99% of administered NMs from the bloodstream are captured by the liver [4]. The liver concentration of intravenously administered NMs is very high even at low injection doses. For example, a quantitative toxicokinetics study showed that TiO_2_-NPs (70 nm) were accumulated mainly in the liver (95.5% of the injected dose after 4 h and 88.9% of the injected dose after 28 d) post intravenous administration (40.13 μg/kg b.w. for the 4-h group and 127.44 μg/kg b.w. for the 28-d group) [19].

Most respiratory-administered NMs deposited in the lung are taken up by macrophages and ultimately dissolve. Only a small amount of respiratory-administered NMs can pass through the ABB and translocate to the blood and liver, even at high doses. In vivo studies reported that the translocation rate of respiratory-administered NMs (i.e., TiO_2_-, Ag-, CeO_2_- and Au-NPs) across the ABB was 0.1%–5% of the initial peripheral lung dose (IPLD) [20, 21] and 0.1%–2% of the administration dose [22–25]. Furthermore, the translocation rate of these NMs to the liver was approximately 0.001%–0.5% of the initial lung dose (ILD) [20, 21, 26] and 0.0023%–0.02% of the administration dose [22–25, 27].

The kinetics of orally administered NMs are more similar to the kinetics of respiratory-administered NMs than to the kinetics of intravenously injected NMs. A very small amount of orally administered NMs can pass through the GIT and translocate to the blood and liver. For example, studies have shown that Au-NPs (1.4–200 nm) accumulated predominantly in the liver post intravenous administration, while liver retention was tenfold higher after intravenous administration than after oral administration [28, 29]. The translocation fraction of orally administered NMs (TiO_2_-, Ag-, and Au-NPs) across the GIT barrier was 0.00%–0.88% of the administration dose [29–31].

### Metabolism and elimination of NMs

NMs escaping the MPS may be taken up by LSECs or pass through the LSEC fenestrae, filter out into the perisinusoidal space and be taken up by hepatocytes and HSCs. NMs are cleared from the body by the MPS, and renal and hepatobiliary systems.

The intracellular trafficking of NMs is summarized as follows. Following uptake, NMs are typically entrapped in vesicular structures, such as endosomes. After endosomal maturation, most endosomal components are delivered to and then degraded in lysosomes. On some occasions, NMs can escape from endosomes and subsequently target intracellular organelles. NMs can be degraded in lysosomes via enzymatic degradation or nonenzymatic degradation. Many xenobiotic metabolizing enzymes with overlapping substrates exist in the lysosomes of liver cells and are usually classified as either phase I enzymes (e.g., CYP450 and flavin-containing monooxygenases) with the main function of oxidizing xenobiotics or phase II enzymes (e.g., glutathione S-transferases, glucuronyl transferases, and sulfotransferases) with the main function of conjugating xenobiotics. Additionally, minor phase I enzymes include epoxide hydrolases, prostaglandin synthetase, dehydrogenases, hydrolases, and proteases. Metal NMs and their oxides may be dissolved to metal ions, which bind to acid radicals, forming metal salt products. For example, Ag-NPs are dissolved in L-929 fibroblast cells and reform insoluble silver species, such as AgCl or AgS [32]. Nonmetallic NMs and their oxides may be degraded into acids. For example, SiO_2_ and Si-NPs are degraded into silicic acid-Si(OH)_4_ and Si(OH_4_), respectively, via successive hydration, hydrolysis, and ion exchange steps [33]. BP-NPs can be degraded into phosphoric acids, such as H_3_PO_4_ [34]. NM degradation products released from lysosomes can target intracellular organelles. Finally, NM degradation products are excreted from cells into the extracellular space via exocytosis in the form of membrane-wrapped vesicles, usually mediated by lysosomes.

**Fig. 2:**
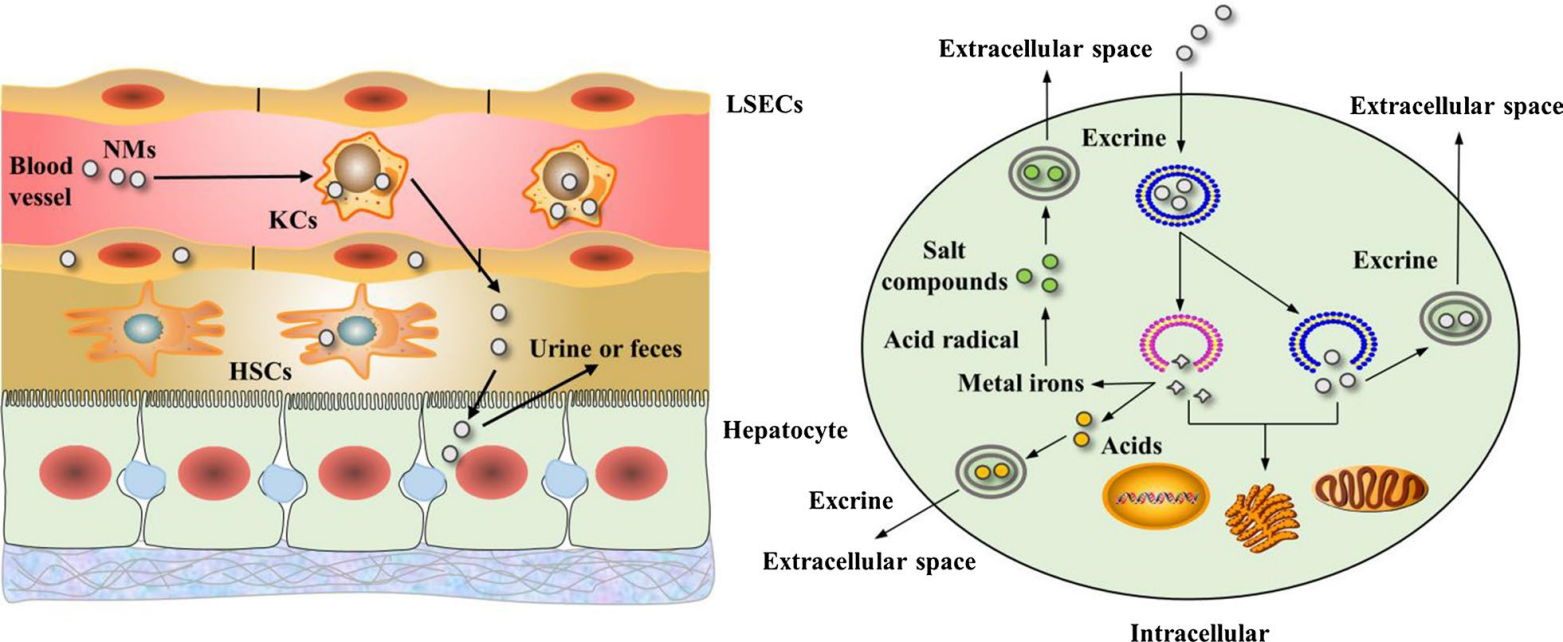
Schematic of the hepatic and intracellular trafficking of NMs. After entering the liver via the portal vein, NMs traversing the hepatic sinusoid are primarily taken up by the MPS, mainly by KCs. NMs escaping from the MPS are taken up by LSECs and may pass through LSEC fenestrae before being taken up by hepatocytes and HSCs. For intracellular trafficking, NMs are typically entrapped in endosomes and degraded in lysosomes. Metal NMs and their oxides may be degraded to metal ions and bound to acid radicals, forming metal salt products. Nonmetallic NMs and their oxides may be degraded to acids. NM degradation products released from lysosomes or NMs that escape from endosomes also target intracellular organelles. Finally, degraded NM products are excreted from cells

A schematic diagram of the hepatic filtration system for NMs and intracellular trafficking of NMs is shown in Fig. 2. The toxicokinetics of representative NMs is summarized in Table 1.

**Table 1:** Toxicokinetics of NMs

Material	Size	Animal model	Administration	Biodistribution	Elimination	References
TiO_2_-NPs	21 nm	F344/DuCrlCrlj rats	10 mg/kg b.w., intravenous injection	Accumulated mostly in the liver (94% of the administration dose) and spleen (2.0%) at 6 h and did not decrease for up to 30 d	Extracted mostly into feces (15 ± 2.9 ng/h), followed by urine (0.27 ± 0.13 ng/h)	[200]
TiO_2_-NPs	70 nm	Wistar rats	40–400 μg/kg b.w., intravenous injection	Highest accumulations in the liver (95.5% of the injected dose) at 1 d, followed by the spleen	Extracted into the GIT then into feces (NM accumulations increase with time) and urine (decrease with time)	[19]
TiO_2_-NPs	8 nm	Wistar rats	40–240 μg/kg b.w., intratracheal instillation	Accumulated mostly in the lung; 1% passed through the ABB (4% of the IPLD) at 1 h	Cleared via the larynx into the GIT and translocated across the ABB into blood	[20]
Au-NPs	1.4–200 nm	Wistar rats	3.1–29 μg/rat, intravenous injection	Accumulated mostly in the liver (from 50 to 99%), followed by the spleen; exhibited a size- and surface charge-dependent distribution	Extracted into feces and urine	[28]
TiO_2_-NPs	40 nm	Wistar rats	30–80 μg/kg b.w., oral application	Accumulated mainly in the skeleton (0.98 ng/g), uterus (0.55 ng/g) and spleen (0.45 ng/g), and a few in the liver (0.09 ng/g) at 7 d	Mostly (0.6% of the applied dose) excreted in feces at 1 h, and 0.05% passed across the GIT barrier	[30]
Au-NPs	1.4–200 nm	Wistar rats	0.8–34.5 μg/rat, intratracheal instillation	Accumulated mostly in the lung (more than 94% at 24 h), and very few reached other organs	Cleared via the larynx and transported into the GIT and feces	[21]
TiO_2_-NPs	20 nm	Wistar rats	1.4 ± 0.5 μg/group, intratracheal instillation	Accumulated mostly in the lung, and 1% of the IPLD reached the liver and kidney	Cleared via the larynx into the GIT and translocation across the ABB, finally excreted in feces and urine	[26]
Ag-NPs	40 nm	Wistar rats	13.5 ± 3.6 μg, intratracheal instillation	Fractions of 0.14/ILD retained in the lung, 0.86 translocated across the ABB, 0.02 each retained in the liver and kidney, 0.48 and 0.22 found in the GIT and feces, respectively	Cleared via the larynx into the GIT or via the blood, liver, and gall bladder into the GIT, then excreted via feces	[168]

## NMs exhibit potential risks for liver disease

The liver is the main target organ of NMs, and liver impairments caused by NMs have been observed in numerous studies. For example, liver impairments such as oxidative stress, inflammatory stress, liver dysfunction, steatosis, fibrosis, DNA damage, and cell death caused by NMs have been widely reported [5–8]. However, the hepatotoxic effects of NMs have not been linked to liver disease, and the toxicity mechanism has not been well demonstrated. This section discusses potential risks for liver diseases induced by various NMs and examines their toxicity mechanisms.

### Nonalcoholic steatohepatitis (NASH)

Nonalcoholic fatty liver disease (NAFLD) is a chronic liver disease that encompasses a wide spectrum of liver diseases that develop progressively from simple steatosis to NASH, fibrosis, and even cirrhosis [35]. Compared with steatosis, NASH is considered a more severe process in which an inflammatory response, liver cell damage, and pericellular fibrosis are involved, and NASH may develop into cirrhosis.

Current studies have shown that several NMs (i.e., Cu-NPs and ZnO -NPs) induce hepatic steatosis, oxidative stress, and cytochrome P450 (CYP450) dysregulation [7, 36, 37]. Further studies have shown that NMs (i.e., ZnO-, CeO_2_-, Au-, CuO- and SiO_2_-NPs) lead to increased lipid synthesis, particularly increased FFA levels [7, 38–41]. Researchers generally presume that NASH is initiated by the overaccumulation of free fatty acids (FFAs) in the liver, attributed to impaired postreceptor signaling induced by insulin resistance (IR). Excessive FFAs lead to lipotoxicity to hepatocytes by inducing ER stress, oxidant stress and inflammasome activation, meanwhile are converted to triglycerides and stored in lipid droplets. IR is initiated by inflammation and cytokine release driven by the NF-κB and MAPK pathways (including ERK, p38 MAPK, and JNK) that lead to the phosphorylation of insulin receptors (IRSs) [42]. Dephosphorylation of IRS1 on serine/threonine residues further initiates the recruitment and activation of PI3K, which phosphorylates serine/threonine moieties of AKT, leading to IR [43]. Importantly, NMs (such as SiO_2_-, TiO_2_-, and ZnO-NPs) induces IR via IRS1 phosphorylation and phosphorylation inhibition of the serine residues of AKT, which is activated by ROS-activated inflammation mediated by the MAPK and NF-κB pathways [44–48]. Based on the above evidence, we postulate that NM exposure may induce NASH by triggering hepatic IR, which is activated by ROS-activated inflammation mediated by the MAPK and NF-κB pathways.

As discussed above, KCs play an important role in NM-induced liver injury because NMs are primarily captured by KCs. The uptake of various NMs (i.e., carbon black, graphene NPs, SIPONs, Cu-, SiO_2_-, and TiO_2_-NPs) leads to macrophage activation and the release of cytokine (e.g., TNF-α, L-1β and IL-18) by activating Toll-like receptors (TLRs), such as TLR 2, 4, and 9, followed by activation of NF-κB, MAPK and JNK signaling [49–54]. TLRs, particularly TLR 2, 4, and 9, regulates inflammation and insulin resistance [55]. Thus, we assumed that NM-activated KCs contribute to IR and lipotoxicity in hepatocytes via the release of cytokines and chemokines. Furthermore, persistent stimuli, such as cytokines, lipid peroxides and molecules, from activated KCs and injured hepatocytes induce HSC activation, leading to fibrogenesis or even cirrhosis.

Thus far, few studies have proven that NMs induce NASH because most of the available toxicological studies on NMs have adopted limited exposure times, most commonly ranging from 1 d to 1 or 2 months, mainly representing acute (up to 14 d), subacute (up to 28 d) and subchronic (up to 90 d) exposure. A study with an exposure time of up to 1 year proved that intratracheally administered MWCNTs trigger a NASH-like phenotype in mouse livers, characterized by inflammation, steatosis, and fibrosis [56]. Thus, chronic (up to 4 months) hepatotoxicological studies of NMs remain a challenge.

**Fig. 3:**
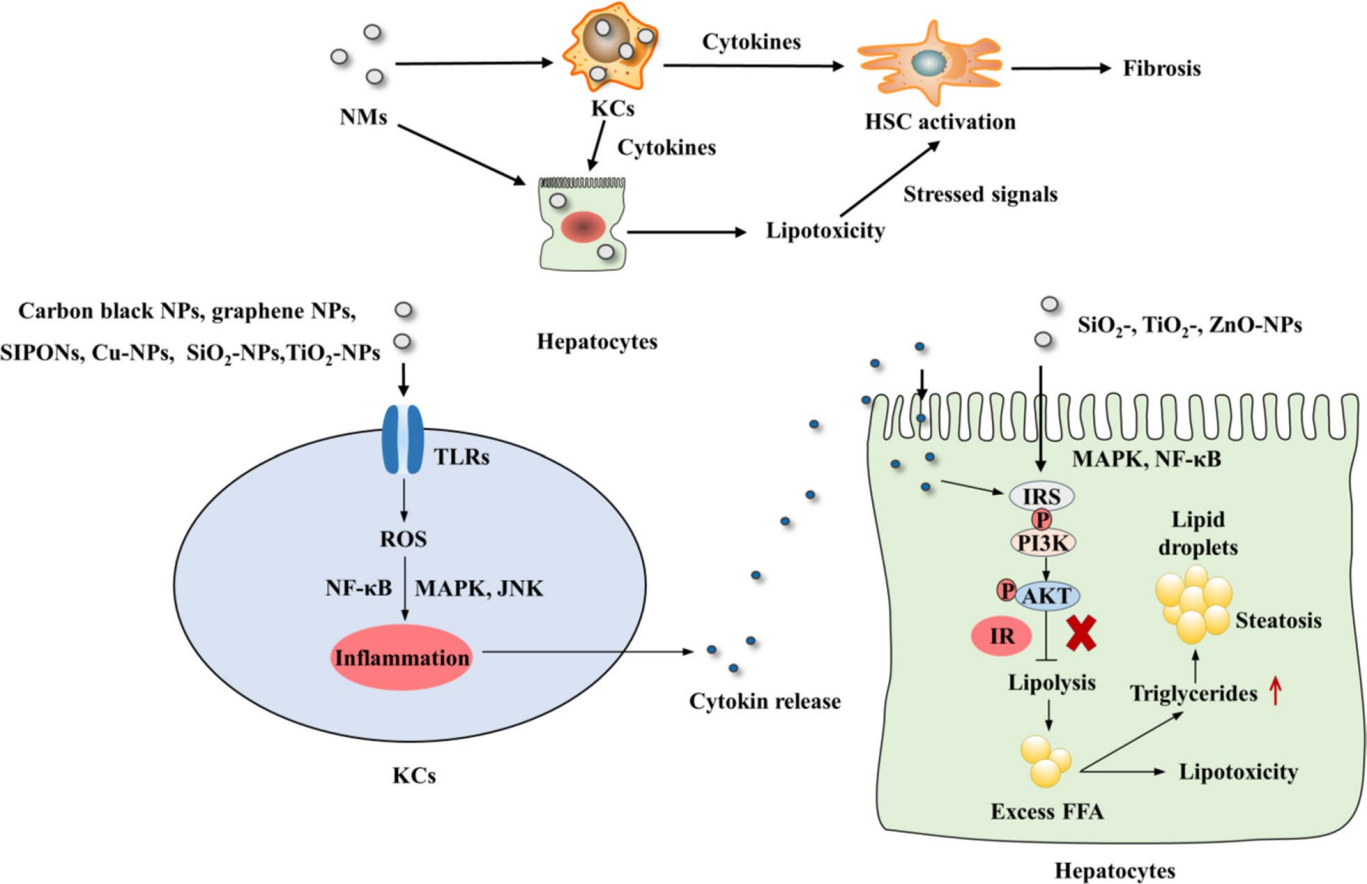
Schematic of the main mechanisms of NM-induced NASH. NM exposure induced persistent stimuli, such as cytokines, lipid peroxides and molecules, from activated KCs, and injured hepatocytes induce HSC activation, leading to fibrogenesis or even cirrhosis. Exposure to NMs (SiO_2_-, TiO_2_-, and ZnO-NPs) induces NASH via hepatic IR, which is triggered by ROS-mediated inflammation activated by the MAPK and NF-κB pathways. The activation of KCs by NMs (carbon black NPs, graphene NPs, SIPONs, Cu- NPs, SiO_2_- NPs, and TiO_2_-NPs) also contributes to IR and lipotoxicity in hepatocytes via the release of cytokines and chemokines

A schematic of the main mechanisms of NM-induced NASH is presented in Fig. 3. Representative studies revealing the contributions of NMs to NASH are summarized in Table 2.

**Table 2:** NM-induced injuries that contribute to NASH

NM	Size	Cells/animals	Dose and exposure time	Effects	References
ZnO-NPs	30 nm	Hens (Jinghong1 strain)	10 mg/kg b.w., oral administration, 4 and 24 w	Caused plasma metabolomic perturbations correlated with hepatic steatosis	[7]
Cu-NPs	23.5 nm	ICR mice	341–1080 mg/kg b.w., oral administration, 48 h	Induced hepatic steatosis	[37]
Au-NPs	20 nm	Wistar rats	0.01 mg/kg b.w., intravenous injection, 2 months	Induced alterations in gene expression related to lipid metabolism in the liver	[38]
CeO_2_-NPs	8 nm	HepG2 cells	6.25–200 μg/mL, 24 h	Induced oxidative stress and increased the content of many lipids, particularly FFAs	[40]
CeO_2_-_,_ SiO_2_-_,_ TiO_2_-_,_ CuO-NPs	5 to > 500 nm	HepG2 cells	3 μg/mL for CuO, 30 μg/mL for CeO_2_ and SiO_2_, and 100 μg/mL for CeO_2_; 72 h	Triggered oxidative stress and increased lipid concentrations	[39]
Au nanospheres and nanostars	40 nm	Wistar rats	1.33 × 10^11^/kg b.w., intravenous administration, 24 h	Triggered oxidative stress and increased FFA levels	[41]
MWCNTs	Diameter: 12.5 ± 2.5 nm, length: 13.0 ± 1.5 mm	C57BL/6 J mice	0.1 mg/mice, intratracheal administration, 1 year	Induced inflammation, steatosis, and fibrosis in the liver	[56]

### Fibrosis

Exposure to several NMs (from 28 d to 9 months) has been shown to induce hepatic fibrosis without inducing hepatic steatosis, such as TiO_2_-NPs [57–59], Cu-NPs [60], SiO_2_-NPs [6], Si-NPs [61, 62], Ag-NPs [63], NiO-NPs [64], and CNTs [56]. Fibrogenesis occurs because of the transformation of HSCs into fibroblasts, which produce ECM faster than it is degraded. During hepatic fibrosis development, the composition of the hepatic ECM transitions from collagen IV and laminin to collagen I and III. Innate immune signaling, particularly that mediated by cytokines and TLRs, contributes to HSC activation.

TGF-β, well acknowledged as the most potent fibrogenic cytokine, excreted by KCs and HSCs, is initially induced by the adaptive immune response (including IL-4, IL-5, IL-13 and IL-17 release). Activation of TGF-β/small mothers against decapentaplegic (SMAD) signaling plays an important role in HSC activation. Activation of TGF-β mediates noncanonical SMAD-independent pathways—e.g., Rho/GTPase, PI3K/AKT, mTOR, and MAPK signaling pathways—which also contribute to HSC activation [65]. Current in vivo studies have shown that NMs—i.e., Cu-, TiO_2_-, and NiO-NPs—induce hepatic fibrosis via the activation of TGF-β/SMAD-dependent and TGF-β/SMAD-independent (i.e., MAPK/WNT, AKT/FOXO3) signaling [58, 60, 64, 66–68]. Thus, based on the evidence above, we postulate that NMs induce HSC activation by stimulating TGF-β/SMAD-dependent and -independent signaling, leading to liver fibrosis.

Activated HSCs express TLRs, including TLR2, TLR3, TLR4, TLR7 and TLR9 [69]. In quiescent HSCs, TLR4 activation upregulates chemokine secretion and the chemotaxis of KCs, as well as downregulates the expression of the TGF-β pseudoreceptor BAMBI to sensitize HSCs to TGF-β-induced activation through a pathway dependent on myeloid differentiation primary response protein 88 (MYD88) and NF‑κB [69]. In vitro studies showed that NMs—i.e., amorphous Si-NPs and Cu-NPs—induce inflammation via activation of the NLRP3 inflammasome and TLR4/MYD88/NF-κB signaling pathway in macrophages and HUVECs [70, 71]. An in vivo study further proved that mesoporous Si-NPs induce liver fibrosis via the TLR4/MYD88/NF-κB signaling pathway [62]. Thus, we postulate that NMs induce HSC activation by activating TLR4/MYD88/NF-κB signaling.

As epigenetic signals, profibrogenic miR‑21, miR‑221/22, and miR‑27 can activate HSCs [69]. In vivo and in vitro studies showed that NMs—i.e., graphene QDs and Ni-NPs—induce miR‑21 regulation in immune cells and liver cells [72, 73]. Additionally, miR‑21 is involved in Ni-NP-induced matrix metallopeptidase (MMP) production (including MMP-2 and MMP-9) [72]. MMP‐2 and MMP‐9 correlate with the severity of inflammation in NASH [74]. Further study shows exposure to Ni-NPs induces miR-21 upregulation, which promotes pulmonary fibrosis, characteristic of TGF-β1 and SMAD2 phosphorylation, and increased COLI and III [75]. Thus, we postulate that NMs induce HSC activation by upregulating miR‑21 expression.

Molecules such as TGF-β, Th2 cytokines (IL-4 or IL-13), and STAT6/STAT3 induce the polarization of M2 macrophages, which are essential for fibrosis. NMs (i.e., SPIONs and MWNTs) exposure to macrophages induce the M1-M2 polarization of macrophages driven by elevated Th2 cytokines (IL-4 and IL-13) [76, 77]. Activation of STAT3 observed in mesoporous Si-NPs induce liver fibrosis [62], and Cu-NPs induce ROS generation, inflammation and CYP450 decrease in the liver [36]. In addition to the finding that NM exposure induces TGF-β activation, we postulate that NMs induce HSC activation by triggering the M1-M2 polarization of KCs via activating IL-4, IL-13, TGF-β and STAT3.

**Fig. 4:**
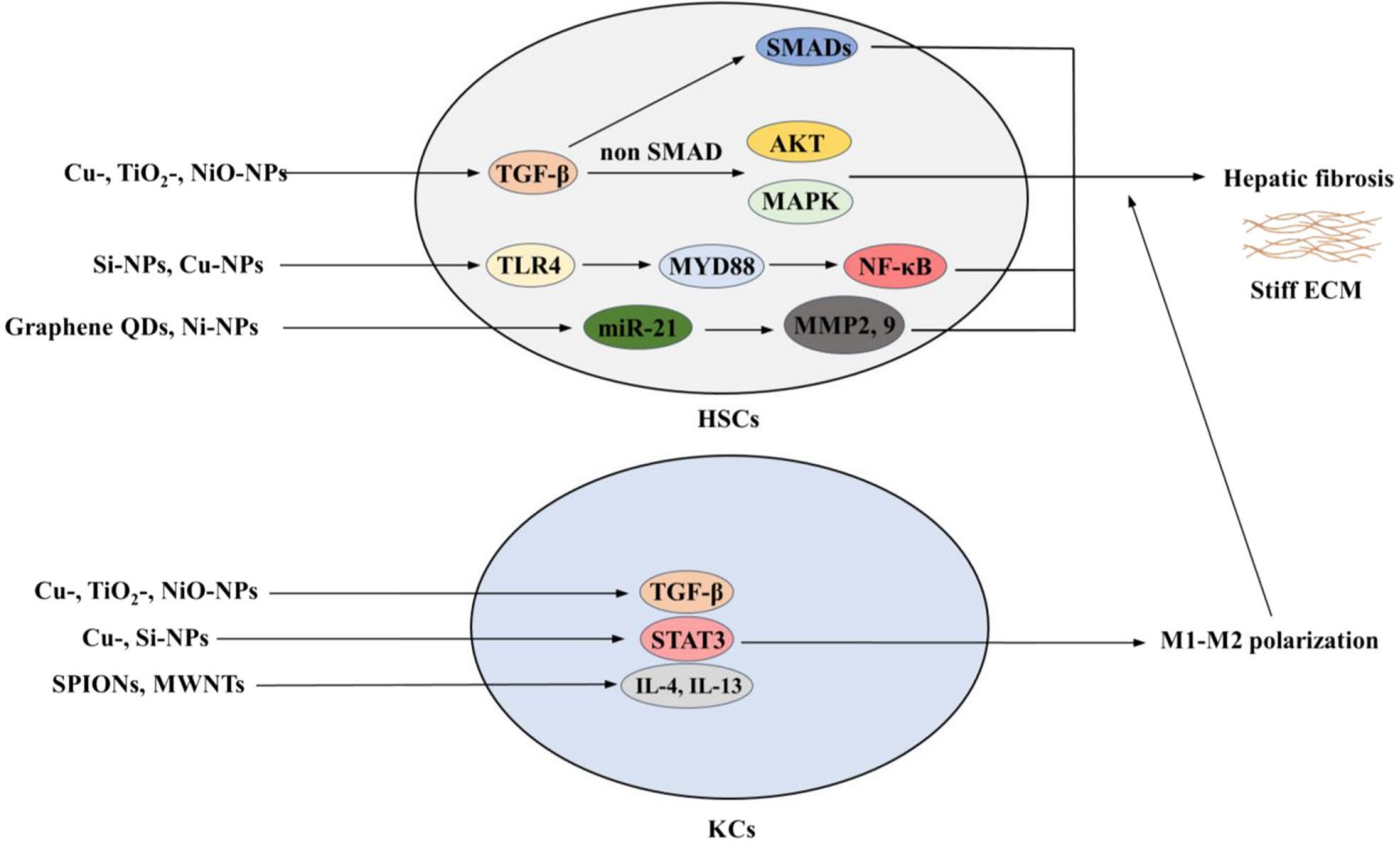
Schematic of the progression and main mechanisms of NM-induced hepatic fibrosis. Cu-, TiO_2_- and NiO-NPs induce HSC activation by stimulating TGF-β/SMAD-dependent and TGF-β/SMAD-independent (i.e., MAPK/WNT and AKT/FOXO3) signaling, leading to liver fibrosis. Si- and Cu-NPs induce HSC activation by activating TLR4/MYD88/NF-κB signaling. Graphene QDs and Ni-NPs induce HSC activation by upregulating miR‑21 expression then activating MMP2, 9 expression. Activation of TGF-β (induced by Cu-, TiO_2_- and NiO-NP), STAT3 (triggered by Cu- and Si-NPs), and IL-4 and IL-13 (induced by SPIONs and MWNTs) induces HSC activation by triggering M1-M2 polarization of KCs

A schematic of the progression and main mechanisms of NM-induced hepatic fibrosis is shown in Fig. 4. Representative studies describing the contributions of NMs to liver fibrosis are summarized in Table 3.

**Table 3:** NM-induced injuries that contribute to fibrosis

NMs	Size	Cells/animals	Dose and exposure time	Effects	References
Si-NPs	64.43 ± 10.50 nm	ICR mice	20 mg/kg b.w., 15 d, intravenous injection	Induced hepatic damage, fibrosis, and TGF-β1/SMAD3 signaling activation	[6]
TiO_2_-NPs	21 nm	Rats	0.5, 1.5, 5, 15, 50, and 150 mg/kg b.w.; intratracheal instillation, 1 month	Induced hepatic profibrotic alterations	[57]
TiO_2_-NPs	5–6 nm	ICR mice	2.5, 5, and 10 mg/kg b.w.; oral administration, 9 months	Triggered hepatic fibrosis and upregulated expression of TGF-β/SMAD/MAPK/WNT pathway-related factors	[58]
Cu-NPs	32.7 ± 10.45 nm	SD rats	100, 200, or 400 mg/kg b.w./day; 28 d, oral administration,	Induced early hepatic fibrotic changes via TGF-β/SMAD signaling	[60]
Ag-NPs	56 nm	SD rats	30, 125, 500 mg/kg b.w., oral exposure, 13 w	Triggered fibrosis in the bile duct	[63]
TiO_2_-NPs	6.5 nm	ICR mice	2.5, 5, 10 mg/kg b.w.; nasal exposure, 9 months	Induced pulmonary toxicity involving reactive free radical-activated TGF-β/SMAD/P38MAPK/WNT signaling	[66]
NiO-NPs	44 nm	Wistar rats, HepG2 cells	0.015–0.24 mg/kg, 9 w, intratracheal instillation	Induced elevated levels of types I and III collagen in the liver and proteins involved in TGF-β1-mediated SMAD pathway	[64]
MWCNTs, SWCNTs, Carbon black	CNTs: 0.3–49 nm × 0.5–10 μm, Silica: 1.6 μm Black carbon: 14 nm	J774A macrophages	2.5 μg/mL, 24 h	MWCNTs and SWCNTs stimulated myofibroblast transformation of macrophages	[201]

### Liver cancer

HCC is the most common liver cancer, accounting for 90% of all hepatic malignancies [78]. The biological mechanisms involved in the progression of HCC include the epithelial-to-mesenchymal transition (EMT), tumor-stromal interactions, the tumor microenvironment, dysregulation of microRNAs and some well-known signaling pathways.

Currently, a few chronic exposure studies have confirmed that NMs (e.g., Si- NPs, ZnO-NPs, TiO_2_- NPs, Ag- NPs, lipid NPs, carbon NPs, and SWCNTs) potentially trigger tumorigenesis or carcinogenesis [79–84]. Additionally, the induction of EMT occurrence by NMs (NiO and TiO_2_-NPs) has been recognized [64, 85]. During the EMT process, epithelial cells lose their cell adhesive properties to each other and a basement membrane and become migratory in nature. The EMT allows cancer cells to avoid death and is a general immune defense. As an external EMT-inducing factor acquired from the microenvironment, TGF-β plays an important role in signals mediating EMT progression, suppressing epithelial differentiation. The TGF-β/WNT, TGF-β/SMAD, TGF-β/MAPK signaling pathways participate in EMT progression [86]. More importantly, a recent study using both in vivo and in vitro components proved that NiO-NPs induce hepatic fibrosis and EMT occurrence through TGF-β1-mediated SMAD pathway activation [64, 85]. Additionally, TiO_2_ and NiO-NP-induced TGF-β/SMAD, TGF-β/WNT and TGF-β/PI3K/AKT/mTOR signaling activation has been reported [58, 60, 64, 66–68]. Therefore, we assume that NMs may induce EMT occurrence by activating TGF-β-mediated WNT, SMAD and MAPK signaling.

Gap junctions are considered an important communication between tumor cells and stromal cells. Loss of Gap junctional intercellular communication (GJIC) and reduced connexin (Cx) expression exacerbate cancer EMT and metastasis [87]. An in vivo study showed that SiO_2_-NP exposure induces GJIC dysregulation involved in Cx43 phosphorylation [88]. Cx43 can be activated by MAPK activation [89]. Importantly, SiO_2_-NP exposure-induced Cx43 phosphorylation is mediated by ERK/MAPK signaling [88]. Therefore, we assume NMs may exacerbate EMT through induce GJIC dysregulation involved in Cx43 phosphorylation via activating ERK/MAPK signaling.

MiR‐21, an important oncogenic mir-RNA, is involved in enhancing EMT, cell proliferation, invasion, migration, apoptosis, and cell elongation in HCC cells by promoting TGF-β/MAPK, TGF-β/SMAD and JNK pathways [90]. Activation of miR‐21 also increases the expression of MMP-2 and MMP-9, known cancer markers involved in invasion, metastasis and angiogenesis of cancer. An in vitro study showed that graphene QDs induce miR‑21 upregulation in immune cells and liver cells [73]. In vivo studies proved that Ni-NPs trigger miR‑21 mediated pulmonary inflammation, fibrosis and protein expression changes (TGF-β1 overexpression and SMAD2 phosphorylation) [75]. Further studies showed that miR‑21 activation is involved in Ni-NP-induced MMP-2 and MMP-9 production [72, 91]. Thus, we postulate that NMs may induce EMT and liver cancer progression by activating miR‑21 expression.

Further tumor development and malignant progression are driven by a dynamic extracellular microenvironment that is impacted by environmental stimuli, immune cell participation, and inflammatory signaling. NF-κB signaling plays an essential role in the inflammation-fibrosis-cancer axis. As discussed above, numerous NMs (such as ZnO, TiO_2_ and SiO_2_-NPs) promote the release of proinflammatory chemokines and cytokines (such as TNF-α, IL-1β, IL18 and IL-6) by activating the ROS-mediated NF-κB pathway [44–47]. Notably, IL-6 production has a mitogenic effect on hepatocytes and can promote HCC progression [92]. Furthermore, within the microenvironment inside a tumor, cancer cells experience different degrees of hypoxia [93]. As a known enhancer of tumor invasion and metastasis, activation of hypoxia-inducible-factor 1 α (HIF-1α) also induces KC activation and cytokine release to contribute to a pro-oncogenic microenvironment. Current studies have shown that NMs, such as Ni-, Co-, TiO_2_-, and ZnO-NPs, induced elevated HIF-1α levels by inducing ROS generation [67, 94–96]. Therefore, we assume that NMs may contribute to cancer development by inducing ROS-mediated inflammation via activating NF-κB signaling and hypoxia via activating HIF-1α signaling.

TiO_2_-NPs exposure induce mutations in rat livers [97]. Further studies showed that NMs—i.e., TiO_2_-, SiO_2_-, Ag-NPs and CNTs—induce mutations by directly binding to DNA and causing altered DNA conformation [98–102] or inducing excess ROS generation, interaction with nuclear proteins, cytoskeleton damage, disturbance of cell cycle checkpoint functions, and release of toxic metal ions from the NM surface [39, 97, 103–105]. Mutant P53 promotes the proliferation and survival of cancer cells. Mutant P53 also induces cancer progression by activating MAPK, TGF-β and HIF1-α signaling [106]. NMs (i.e., Si-NPs and CNTs) induce cell cycle disruption and increase mitotic spindles [105, 107–109]. Furthermore, P53 inhibition was observed during malignant transformation induced by NMs (i.e., Si-NPs and CNTs). In addition to the evidence that exposure to NMs (such as Si-NPs and CNTs) induces activation of these signals (MAPK, TGF-β and HIF1-α) [6, 56, 88, 110], we speculate that P53 mutation may be involved in NM-induced liver cancer progression by activating MAPK, TGF-β and HIF1-α signaling.

Even if NMs have carcinogenic potential, NM-induced carcinogenesis or tumorigenesis has been demonstrated in few studies. As discussed above, the major drawback of current toxicological studies on NMs is the limited exposure time.

**Fig. 5:**
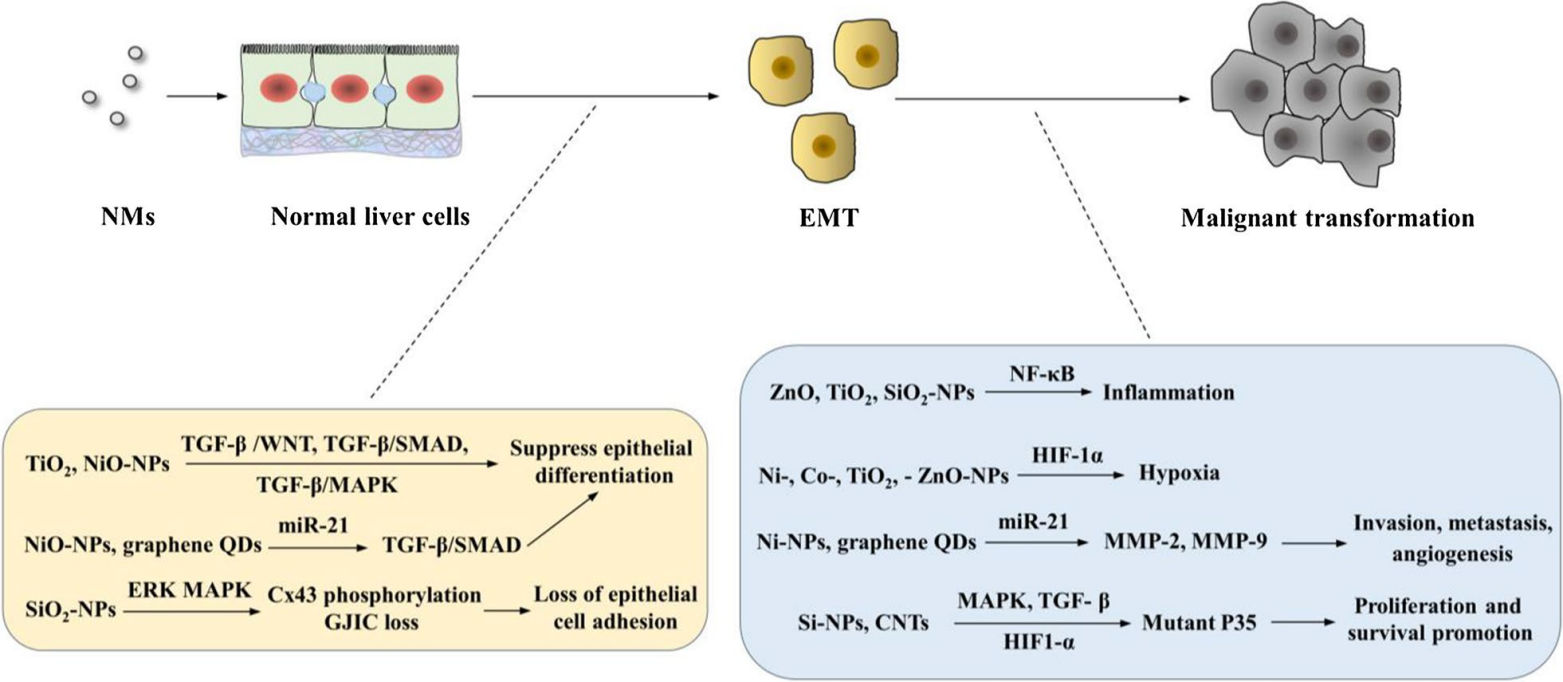
Schematic of the signaling pathways involved in NM-induced liver cancer. TiO_2_ and NiO-NPs induce EMT occurrence by activating TGF-β activated WNT, SMAD and MAPK signaling. NiO-NPs and graphene QDs trigger EMT by activating miR-21 mediated TGF-β/SMAD signaling. SiO_2_-NPs exacerbate EMT by inducing GJIC dysregulation involved in Cx43 phosphorylation via activating ERK/MAPK signaling. NMs contribute to cancer development by inducing inflammation via activating NF-κB signaling (ZnO-, TiO_2_-, and SiO_2_-NPs), and hypoxia via activating HIF-1α signaling (ZnO-, TiO_2_-, Ni-, and Co- NPs). Ni-NPs and graphene QDs induce liver cancer progression via activating miR‑21 expression, thus upregulating MMP- and MMP-9 expression. P53 mutation is involved in NM (Si-NPs and CNTs) induced liver cancer progression by activating MAPK, TGF-β and HIF1-α signaling

A schematic diagram of the signaling pathways involved in NM-induced liver cancer is shown in Fig. 5. Representative studies revealing the potential contributions of NMs to liver cancer are summarized in Table 4.

**Table 4:** NM-induced injuries that may contribute to cancer

NMs	Size	Cells/animals	Dose and exposure time	Effects	References
TiO_2_-NPs	5–6 nm	ICR mice	1.25, 2.5, and 5 mg/kg b.w., nasal exposure, 9 months	Caused pulmonary tumorigenesis	[82]
Lipid-NPs	150 nm	Kunming mice	16.5 mg/kg b.w., oral administration, 5, 10, 15, and 20 w	Enhanced the oncogenic effects of diethylnitrosamine and resulted in liver cancer in mice	[84]
SWCNTs	Width: 0.1–1 μm, length: 0.8–1.2 nm	BEAS-2B cells	In vitro: 0.02 μg/cm^2^, 12 and 24 w; in vivo: 2 × 10^6^ B-SWCNTs and passage-control cells, subcutaneous injection, 12 and 14 d	Induced malignant transformation and tumorigenesis	[81]
SWCNTs	Width: 0.8 to 1.2 nm, length: 0.1 to 1 μm	BEAS-2B cells	0.1 μg/mL, 6 months	Triggered gene expression alterations related to oncogenic potential	[79]
SWCNT	Width: 0.1–1 μm, length: 0.8–1.2 nm	SAEC cells and BEAS-2B cells	In vivo: 2 × 10^6^ SWCNT-treated SAEC and BEAS-2B cells, subcutaneous; in vitro: 0.1 μg/mL, 6 months; injection, 21, 28, and 35 d	Induced the transformation of human lung epithelial cells to stem-like cells with malignant properties	[80]
Si-NPs	57.66 ± 7.30 nm	BALB/c nude mice, BEAS-2B cells	In vivo: 1 × 10^7^ Si-NP-treated cells, subcutaneous injection, 3–4 w; In vitro: 5 μg/mL, 18 w	Induced malignant transformation and tumorigenesis of human lung epithelial cells via P53 signaling	[83]

### Metabolic disorders

#### Iron accumulation disorders

The liver is the main site of iron storage and plays a dominant role in iron homeostasis regulation by secreting most proteins involved in iron metabolism, such as hepcidin and transferrin.

Iron homeostasis dysregulation can result from chronic liver diseases, such as hepatitis, NAFLD and hepatic cancer. Because NM exposure likely induces these hepatic diseases, they may induce iron homeostasis dysregulation. Hepcidin overexpression leads to degradation of the receptor ferroportin, resulting in a block in iron efflux and iron overload. Hepcidin secretion can be stimulated by inflammation through IL-6, IL22, STAT3 signaling activation and the ERK/MAPK and BMP/SMAD pathways [111]. Hepcidin overexpression occurs during the progression of liver diseases, such as hepatitis, NASH and hepatic cancers. Exposure to NMs, such as Si-, NiO-, SiO_2_-, and Cu-NPs, induce liver injury by activating the ERK/MAPK, SMAD, IL-6, STAT3 signaling pathways [44, 60, 62, 64, 85, 112]. Thus, NM exposure likely induces iron overload by stimulating hepcidin secretion and activating the ERK/MAPK, SMAD, IL-6, STAT3 signaling pathways.

Additionally, excessive exposure to iron oxide NPs (IONPs) directly leads to iron overload. IONP exposure-induced iron overload and liver toxicity have been reported [113]. The toxic mechanism of IONPs mainly results from ROS generation and iron ion release. In vitro models showed IONP exposure induces increased intracellular iron concentrations [114, 115]. The transition of iron from Fe^3+^ to ferrous Fe^2+^ in endosomes can trigger the Fenton reaction (Fe^3+^ + O_2_=Fe^2+^ + O_2_^−^), resulting in oxygen radical generation. After degradation in endosomes, Fe^2+^ is then released into an iron pool in the cytoplasm and excess iron is stored in ferritin. Correspondingly, studies showed that the degradation of IONPs in the endosome of KCs leads to increased secretion of ferritin and cytokine release [116, 117].

Finally, iron overload-induced excess ROS generation further induces ER stress, inflammation, and mitochondrial stress [118], resulting in an increased risk of hepatitis, NAFLD, fibrosis, cirrhosis and hepatic cancer.

#### Copper accumulation disorders

The liver is the main site of copper metabolism. High concentrations of copper NMs induce hepatic injuries, such as hepatic steatosis, inflammation, ROS generation, fibrosis and CYP450 depression [37, 119, 120]. Cu-NP exposure leads to liver toxicity mainly because of the ability of Cu-NPs to generate ROS, release copper ions and show high affinity for enzymes and proteins. Because copper is a highly soluble NM, the dissolution of copper ions contributes markedly to the toxicity of copper NPs. For example, in fetal calf serum-free DMEM, the solubility of copper ions released from CuO-NPs was 66% [121]. In the presence of superoxide or reducing agents, Cu^2+^ is reduced to Cu^+^, which catalyzes the formation of hydroxyl radicals.

Current studies have revealed that Cu-NP exposure leads to CYP450 inhibition in rats [36, 122, 123]. Because CYP450 enzymes are responsible for the metabolism of fatty acids and xenobiotics, their inhibition can reduce the hepatic biotransformation of NMs, aggravating the toxicity of NPs. Further studies showed that Cu^2+^ released from Cu-NPs both oxidized and bound to some amino acid residues of the CYP450 monooxygenase, resulting in decreased CYP450 levels [124]. The hepatic CYP450 levels are activated by NF-κB [125] signaling and are closely related to MAPK [126] and STAT3 signaling [127]. Additionally, aryl hydrocarbon receptor (AHR), constitutive androstane receptor (CAR), and pregnane X receptor (PXR) are regulators of CYP450s. More importantly, current studies have shown that Cu-NPs induce CYP450 expression decrease via ROS-mediated activation of NF-κB, MAPK and STAT3 signaling pathways [36, 122, 128]. Additionally, elevated mRNA levels of PXR, CAR and AHR were detected in Cu-NP treated rats [122]. Based on the above studies, we speculate that exposure to Cu-NPs induces CYP450 depression, which aggravates the toxicity of NMs via ROS mediated activation of NF-κB, MAPK and STAT3 signaling and overexpression of PXR, CAR and AHR.

**Fig. 6:**
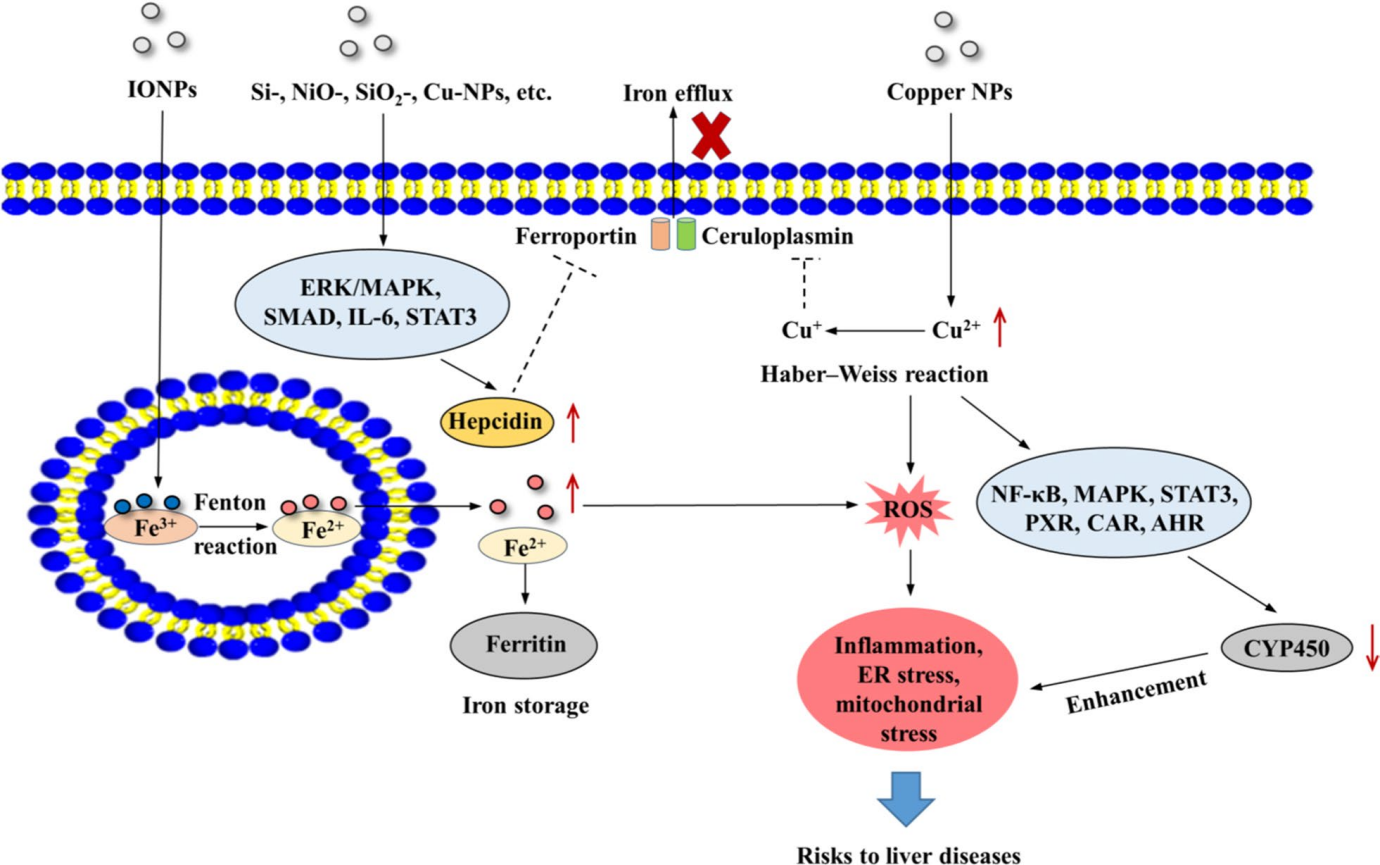
Schematic of NM-induced iron and copper toxicity. NMs, such as Si-, NiO-, SiO_2_- and Cu-NPs, induce iron overload by stimulating hepcidin secretion and activating ERK/MAPK, BMP/SMAD, IL-6, STAT3 signaling. Hepcidin overexpression leads to ferroportin reduction, resulting in iron efflux obstruction. Additionally, IONP exposure leads to liver toxicity by inducing ROS generation via triggering the Fenton reaction. Additionally, IONP exposure leads to increased secretion of ferritin. High concentrations of copper NMs induce CYP450 depression, which aggravates the toxicity of NMs by ROS-mediated activation of NF-κB, MAPK, and STAT3 signaling, as well as overexpression of PXR, CAR and AHR. Cu^2+^ released from copper NPs is reduced to Cu^+^, which catalyzes the formation of hydroxyl radicals. Copper overload also results in the depletion of ceruloplasmin, leading to decreased iron efflux and iron overaccumulation. Finally, iron and copper overload-induced excess ROS generation further induces ER stress, inflammation, and mitochondrial stress, resulting in an increased risk of liver diseases

Copper also participates in the synthesis of ceruloplasmin, promoting iron efflux in concert with ferroportin by oxidizing Fe^2+^ to Fe^3+^ [129]. Copper overaccumulation induces the depletion of ceruloplasmin. Thus, Cu-NP exposure may induce decreased iron efflux and iron overload via depletion of ceruloplasmin. A schematic of NM-induced iron and copper toxicity is shown in Fig. 6.

## Toxicokinetics and toxicity of NMs in livers of susceptible individuals

### Subjects with liver disease

Studies have shown that the livers of subjects with liver disease, such as hepatitis or NASH, are more vulnerable than normal livers to exposure of NMs—i.e., Au-NRs, PEG-Au-NPs, and MWCNTs—because of the increased burden on liver cells [130–132]. The livers of subjects with liver disease are more vulnerable than normal livers likely because of increased NM accumulations in the liver.

Morphological and/or functional alterations in liver cells, as well as hepatic microcirculatory disorders, occur under hepatic pathological conditions, leading to altered NM distribution. For example, cancerous tissue and inflammatory reactions induce the release of proinflammatory cytokines and vascular endothelial growth factor (VEGF), resulting in increased LSEC fenestration—i.e., a loss of cellular integrity—triggering a gap between LSECs. This event allows NMs to extravasate from the blood system into the inflamed and cancerous tissue. By contrast, diseases such as liver fibrosis and cirrhosis are associated with decreased cellular distribution of NMs because the capillarization of LSECs causes the loss of LSEC fenestrae, appearance of a basement membrane, and increased ECM production, leading to decreased cellular distribution of NMs [69]. However, studies have proven that HSCs capture more NMs in fibrosis livers than in normal livers [133, 134]. The cause may be that, during fibrosis, HSCs proliferate 10–20 times and squeeze through LSEC fenestrations, leading to an increased chance of contact with blood. More importantly, during the progression of liver diseases, liver injuries—i.e., hepatocyte death and dysfunction—and increased energy consumption lead to decreased hepatic metabolism and clearance of NMs, which play a dominant role in facilitating disease progression. Therefore, the liver shows much higher plasma accumulation of various drugs in subjects with hepatitis, NASH, cirrhosis, cholestasis, and liver cancer than in normal subjects [135].

### Aging and young subjects

Because of the senescence changes in aging subjects and immature physiological structures and functions in young subjects, they are more susceptible than adult individuals to injuries. Studies have proven the age-dependent deposition or translocation across physical barriers of NMs [136, 137]. Additionally, in vivo and in vitro studies have shown that senile subjects and cells are more susceptible to NM exposure than young ones, followed by adult ones [138–140]. For example, Chen et al. [141] investigated SiO_2_-NP inhalation-induced toxic effects in rats and showed that the risk of pulmonary damage decreased in the order of old (20 months) > young (3 w) > adult (8 w) rats and that a risk of cardiovascular disorder was observed only in older animals.

### Obese subjects

Current studies have shown the livers of obese individuals are susceptible to NM exposure. Because the expansion of adipose tissue in obese subjects diminishes their ability to store excess energy, adipocyte dysfunction and enhanced lipolysis occur [131]. NM exposure further enhances obesity-driven lipolysis, resulting in increased levels of circulating FFAs and lipotoxicity, which enhance the development of hepatic diseases. For example, studies have revealed that NM exposure promotes obesity-driven liver diseases by inducing ROS generation, IR, proinflammatory activation of KCs and fatty acid oxidation [142, 143].

### Subjects of both sexes

Male and female individuals differ in many aspects regarding the vulnerability to NMs and other stressors. Differences between sexes are attributed to sociocultural factors; exposure; genetic, molecular, and biochemical factors; body size and composition; and hormonal and reproductive factors. For example, after Ag-NP exposure, differentially expressed hepatic genes in male rats were involved in diabetes and metabolism, but metabolism and cell signaling changes were detected in female rats [144]. An in vitro study showed that female human amniotic stem cells captured more QDs than male cells, but male fibroblasts captured more QDs than female fibroblasts [145]. The differences in cell uptake could be attributed to variations in secreted paracrine factors and the cytoskeleton. More details on the role of sex in the toxicity of NMs or other xenobiotics are presented in other reviews [146, 147].

Although NMs potentially trigger more severe toxic effects in susceptible livers, the current studies on NM-induced toxicity in susceptible liver models are still very limited. To reduce the gap between laboratory discoveries and safe application of NMs in various fields, overlooked factors that influence nanotoxicity must be examined. Hopefully, to better evaluate the safety of NMs in various fields, further studies will consider hepatic susceptibility-related factors.

## Factors affecting the toxicokinetics and toxicity of NMs

The toxicokinetics and toxicity of NMs depend on the PCM characteristics of NMs (e.g., size, shape, charge, dissolution, agglomeration/aggregation, hydrophobicity/hydrophilicity and functionalization) and interactions with biological systems.

### Concentration

Various studies have proven that NM exposure causes dose-dependent liver toxicity in vivo and in vitro [8, 148, 149]. Hepatocytes capture elevated NMs with increasing NM concentrations; however, when the concentration is exceeded by a certain amount, MPS uptake plays an important role because NMs at high concentrations are primarily uptake by MPS. For example, a PBPK model predicted that, with an increasing administration dose, the amount of Ag-NPs captured by the liver increased and was highest among organs in the highest dose group [150]. Furthermore, a PBPK model deduced that the MPS uptake rates were 0%, 25%, and 100% of the original rate in the low-dose (300 mg/kg), moderate-dose (500 mg/kg) and high-dose (1000 mg/kg) groups of intravenously administered Ag-NPs, respectively [150]. Kim et al. [151] proved that the uptake of Ag-NPs by the MPS is negligible below a blood Ag concentration of 180 ng/g.

### Size

The NM size plays a dominant role in their toxicokinetics and toxicity. Small NMs are more toxic than large NMs because of their higher reactive surface areas and ability to pass the biological barrier.

Large particles (submicron and micron scales) have a low likelihood of crossing the liver barrier because they show more efficient uptake by the MPS [5]. By contrast, small NMs can pass through the hepatic barrier more efficiently than large NMs because the diameter of LSEC fenestrae is reported to be 50 to 150 nm [152]. For example, an in vivo study confirmed that intravenously injected 18-nm Au-NPs were captured by KCs, LSECs and hepatocytes [28]. By contrast, after intravenous injection, 100- and 200-nm Au-NPs were observed only within the vessels of liver lobules [153]. Similarly, another in vivo study showed that Ag was present in KCs, LSECs and hepatocytes in 10-nm-AgNP-treated mice; in portal endothelial cells, LSECs and KCs in 40-nm-AgNP-treated mice; and mostly in KCs in 100-nm-Ag-NP-treated mice [154]. Additionally, smaller NMs enter cells primarily through receptor-mediated or independent endocytosis, which occurs in all types of liver cells [155, 156]. However, large NMs are mainly taken up by KCs via phagocytosis.

Small NMs pass through the ABB [21, 137, 157] and GIT barrier [29] more efficiently than large NMs. For example, NMs with hydration diameters < 6 nm can pass through the ABB rapidly and enter lymph nodes and the bloodstream [158]. Furthermore, potential clearance from the body depends largely on the NM size. NMs larger than 100 nm are cleared only through the liver and MPS, but NMs with smaller sizes (< 6 nm) can penetrate the renal tubules and enter the urine [4]. Larger NPs showed faster degradation than smaller NPs [159], likely because of their more efficient capture by the MPS.

Higher reactive surface areas of small NMs lead to higher toxicity. For example, the small (10 nm) Ag-NPs lead to higher silver tissue accumulation, resulting in overt acute toxic effects compared with the larger NPs (40 and 100 nm) because small Ag-NPs present with higher silver iron dissolutions resulting from their higher reactive surface areas [154].

### Agglomeration/aggregation

Agglomeration/aggregation affects NM uptake amounts and pathways. In vivo and in vitro studies have reported that agglomeration decreased the uptake of NMs (including TiO_2_-, Ni-, Si-, and Au-NPs) [155, 160–162], likely due to a reduced NM surface area. However, large agglomerates of NPs induce greater cellular uptake to macrophages than small agglomerates [163] because large agglomerates are more suitable than small agglomerates for phagocytosis [164].

Agglomeration/aggregation also substantially affects NM toxic behavior. Most studies have reported that agglomeration increases NM toxicity [155, 160–162], while other studies have revealed that agglomeration decreases NM toxicity [163, 165]. For example, oral gavage of large agglomerates of TiO_2_-NPs (primary size: 117 nm) induces higher pulmonary responses in mice than oral gavage of small agglomerates [163]. However, Noël et al. [166] showed that inhaled exposure to large agglomerate aerosols of TiO_2_-NPs (primary size: 5–50 nm) does not induce more severe toxic effects than small agglomerate aerosols. Other factors (i.e., disperse/culture medium, NM concentration, primary size of NMs, exposure time, exposure route and cell type) also affect the agglomeration profile and are responsible for the observed contradictory toxic effects of NM agglomeration. Interestingly, Noël et al. [166] proved that large agglomerate aerosols of TiO_2_-NPs induce an acute inflammatory response, while small agglomerate aerosols produce oxidative stress damage and cytotoxicity.

### Dissolution

Dissolution, as a measure of biodurability, is dependent on the PCM properties (e.g., size and surface area) of NMs and suspension medium (e.g., pH, ionic strength, and ion concentration). Biodurability has a potential influence on the toxicity of NMs.

According to current studies, the toxicity of highly soluble NMs mainly results from released iron, while that of poorly soluble NMs are mainly attributed to the NMs. For example, Zn^2+^ release largely contributes to ZnO-NP-induced toxicity [167]; the CuO-NP-induced toxic effect originated from both the released irons and CuO-NPs; and the toxic effects of ZrO_2_-, Fe_2_O_3_-, CeO_2_-, Ag- and NiO-NPs were mainly related to the metal NPs [136, 168–171].

Dissolution of NMs is an important clearance route for translocation [172]. Thus, highly soluble NMs (i.e., ZnO-NPs) show more rapid and efficient translocation than NMs with low solubility (e.g., CeO_2_-, TiO_2_-, and Au-NPs) because of the reduced dimensions of highly soluble NMs [136, 168, 173]. For example, in vivo data showed that an SiO_2_ coating attenuated the dissolution of ZnO-NPs, decreasing their translocation rate across the ABB from 7.4% to 6.7% [174]. ZnO-NPs exhibit a much higher translocation rate across a lung epithelial cell monolayer than CeO_2_-NPs in vitro [173]. Additionally, biodurable NMs are more likely to be taken up and eliminated by the MPS. By contrast, non-biodurable NMs are captured and eliminated through the hepatic and renal pathways.

### Shape

NMs come in various shapes—i.e., spherical, rod-like, needle-like, flake-like, fiber-like and horn-like. The shape of NMs determines their cellular uptake (endocytosis and phagocytosis), impacting their toxicity. Non-spherical NPs, particularly needle-shaped and rod-like analogous NMs, are more toxic because they more easily puncture the cell membrane to be subsequently taken up by cells [175]. Additionally, CNTs induce a greater hazard risk than other NMs mainly because of their fiber-like shape. The long fiber-like shape of CNTs leads to incomplete enclosure by macrophages, leading to frustrated phagocytosis and clearance. For example, a recent study showed that CNTs exhibit more toxicity over single-walled carbon nanohorns by triggering more lysosome stress, protein expression changes, cell death, pyroptosis, necrosis and apoptosis in macrophages [5]. Additionally, the pulmonary fibrotic effects of CNTs are well established and even trigger a lung fibrotic response at a very early stage after exposure [176–178]. Furthermore, the carcinogenic potential, such as the level of centrosome fragmentation, mitotic spindle damage and aneuploidy after exposure to MWCNTs, is similar to the effects of the known carcinogen vanadium pentoxide [108].

### Hydrophobicity/hydrophilicity

Hydrophobicity/hydrophilicity determines the NM biodistribution, cell uptake model, interaction between NMs and the cell membrane, and ability to pass the biological barrier.

For large NMs, membrane wrapping (i.e., endocytosis) has been recognized as the main cell uptake pathway, while small NMs with certain PCM properties tend to penetrate the cell membrane independently. Studies have demonstrated that the hydrophilic and hydrophobic NMs exhibit varied cell membrane interactions and cell uptake [179, 180]. A computational model showed that hydrophobic NMs (10–40 nm) undergo penetration, leading to deformation and heterogeneity in the distribution of lipid molecules in the bilayer [181]. By contrast, hydrophilic NMs can be adsorbed on the surface of the bilayer rather than entering the core of the membrane, triggering membrane wrapping. In vitro studies further verify the results of computational models. Hydrophilic NMs—i.e., metal NMs—are generally taken up by cells via wrapping [155, 182, 183]. By contrast, hydrophobic NMs, such as fullerenes and graphene, undergo penetration into cells [181, 184].

Studies also proved the influence of hydrophobicity/hydrophilicity on NM biodistribution and the ability to pass the biological membrane. For example, hydrophilic TiO_2_-NPs were freely distributed in the cytoplasm after contact with the buccal mucosa, whereas their hydrophobic counterparts were engulfed in the vesicular structures of the buccal mucosa [185]. Additionally, hydrophilic NMs exhibited priority to pass the blood–brain barrier and enter the cerebrospinal fluid [186].

### Charge

The surface charge of NMs affects biological aspects such as biodistribution, cell uptake mode and plasma protein binding.

Positively charged NMs perturb the continuity of the plasma membrane and trigger more severe toxic effects than negatively charged NMs because of their interaction with the negatively charged surface of cells. For example, positively charged polystyrene NPs rather than negatively charged NPs induce apoptotic signaling to alveolar cells [187]. Studies have also proven that the surface charge determines the biodistribution of NPs, as well as the internalization and uptake mechanisms of cells. For example, Tang et al. [188] reported most of the negatively charged CdSe/ZnS core–shell QDs would deposit in the liver, but the positively charged QDs are detected in the lung. Nagy et al. [189] reported the positively charged CdS/ZnS-QDs were more likely to be phagocytosed by macrophages, and the negatively charged QDs were most likely to be incorporated. Surface charge also determines protein absorption, which affect corona formation. For example, an electrostatic model showed that, under neutral conditions, the local pH around positively and negatively charged NPs become very high and low, respectively, with a profound impact on the protein charge, hydration and affinity to the nanodiamonds [190]. Furthermore, surface charge affects the NM agglomeration/aggregation state through impacting the protein adsorption. For example, positively charged Au-NRs aggregate extensively when exposed to excised human skin compared with neutrally and negatively charged nanorods [191]. The aggregation of positively charged Au-NRs is attributed to the adsorption of proteins released from skin.

### Functionalization

Because NMs exhibit toxicity to living systems, surface modification of NMs is generally applied to reduce their toxicity effects and alter their toxicokinetics. Studies have confirmed that the functionalization of NMs such as polyethylene glycol (PEG), polyvinylpyrrolidone (PVP), and pluronic, largely decreases the toxicity and improves the biocompatibility of NMs. For example, PEG is mainly used for the passivation of NMs to obtain neutral surface charge, which prevents nonspecific protein absorption, immunological reaction and accumulation in the MPS [152]. Studies have shown that PEG-coated NPs induce fewer toxic effects than positive or negative NPs and reduce the sequestration of NPs by the MPS, thus increasing the circulation time [159, 188]. Lee et al. [192] reported that PEG-coated SWCNTs showed decreased adsorption of proteins, affecting corona formation and altering their toxicokinetics [193]. PVP-coated NMs also show altered distribution and decreased toxicity. For example, a recent study reported that PVP-coated MoS_2_ nanosheets show increased biodegradation and clearance [194]. Additionally, Wang et al. [195] reported PVP‐coated Ag-NPs with the same particle size cause less DNA damage and chromosomal aberrations than uncoated NPs to hepatocytes.

### Corona

After entering the internal environment, NMs are coated with coronas, such as those acquired in the serum, lung and GIT. Corona formation determines NM toxicity because it affects the PCM properties of NMs—i.e., aggregation/agglomeration, dissolution and hydrophilicity—which affect the cell uptake model and toxicokinetics of NMs. For example, the corona was reported to reduce the aggregation of Au-NPs and Au-NRs, resulting in the processing of 15-nm Au-NPs via the endocytosis pathway, a change in the processing of 45-nm Au-NPs by hepatocytes and gastric epithelial cells from receptor-mediated endocytosis to the macropinocytosis pathway, and a change in the processing of Au-NRs (33 × 10 nm) from receptor-mediated endocytosis to clathrin- and caveolin-independent pathways under non-fetal bovine serum-coated conditions [155]. Studies revealed corona formation also influences the dissolution and hydrophilicity of NMs, which affect the cell uptake of NMs [196, 197]. Additionally, corona formation can alter the recognition of NMs by the MPS because some components of the corona (i.e., albumin) lead to reduced phagocytosis [198], while other components (i.e., IgG and pulmonary surfactant proteins) result in increased phagocytosis [199].Reference: Kindly check whether the inserted [Page range] for references [16, 18, 36, 44, 62, 86, 88, 117] are appropriate.Yes, they are appropriate.

## Conclusions and outlook

The liver is the main organ targeted after exposure to NMs. Thus, the safety and effects of NMs in the liver are a research focus. This review highlights the possibility that NMs induce hepatic diseases, including NASH, fibrosis, liver cancer, and metabolic disorders, and explores the underlying intrinsic mechanisms based on the current understanding of NM toxicokinetics. Additionally, the toxicokinetics and potential risks of NMs in susceptible livers are summarized. The influences of the PCM properties of NMs on their toxicokinetics and toxicity are also explored. We hope to provide guidance for further toxicological studies on NMs that will be important to further develop NMs for applications in various fields.

Although some evidence of NM-induced liver diseases and intrinsic mechanisms is available, a wide gap between current knowledge and unexplored facts persists. The currently available studies have verified certain primary hepatotoxic effects of NMs; these toxic effects have not been linked to liver disease, and the toxicity mechanism remains unclear. Thus, in-depth studies on NM-induced liver diseases and the intrinsic mechanisms with validation of effective methodologies and well-designed experimental models are needed. Additionally, most of the exposure times adopted in current studies represent acute, subacute and subchronic toxicity. Chronic hepatotoxicity studies of NMs are needed, particularly to observe NM-induced NASH, liver fibrosis and cancer. Furthermore, NMs exhibit various distributions and exert enhanced toxic effects in susceptible livers, and this issue has been ignored in current toxicity studies. Therefore, further studies should consider susceptibility-related factors (including subjects with liver disease, obese individuals, aging and young individuals, and individuals of both sexes) to better evaluate the safety of NMs.

## Data Availability

Databases/repositories and materials are not applicable in this review.

## Abbreviations

NMs: Nanomaterials
NPs: Nanoparticles
NASH: Nonalcoholic steatohepatitis
NAFLD: Nonalcoholic fatty liver disease
LSECs: Liver sinusoidal endothelial cells
KCs: Kupffer cells
HSCs: Hepatic stellate cells
BECs: Biliary epithelial cells
HPCs: Hepatic progenitor cells
MPS: Mononuclear phagocyte system
ECM: Extracellular matrix
ABB: Air-blood barrier
GIT: Gastrointestinal tract
PBPK: Physiologically based pharmacokinetics
PCM: Physicochemical and morphological
ILD: Initial lung dose
IPLD: Initial peripheral lung dose
BP: Black phosphorus
QDs: Quantum dots
NRs: Nanorods
CNTs: Carbon nanotubes
FFA: Free fatty acid
IR: Insulin resistance
IRS: Insulin receptors
PI3K: PI 3-kinase
STAT3: Signal transducer and activator of transcription 3
SMAD: Small mothers against decapentaplegic
TLRs: Toll-like receptors
ER: Endoplasmic reticulum
MMPs: Matrix metallopeptidases
MWCNTs: Multiwalled carbon nanotubes
SWCNT: Single-walled carbon nanotubes
SPIONs: Iron oxide nanoparticles
CYP450: Cytochrome P450
HCC: Hepatocellular carcinoma
EMT: Epithelial-to-mesenchymal transition
TGF-β: Transforming growth factor β
AHR: Aryl hydrocarbon receptor
CAR: Constitutive androstane receptor
PXR: Pregnane X receptor
AHR: Aryl hydrocarbon receptor
CAR: Constitutive androstane receptor
PXR: Pregnane X receptor
PEG: Polyethylene glycol
PVP: Polyvinylpyrrolidone
